# Identification of an in-frame insertion in ACKR1 in five individuals from Agri community, India

**DOI:** 10.1016/j.htct.2025.106075

**Published:** 2025-10-30

**Authors:** Shaikh Roshan, Solanki Prakash, Ghosh Kanjaksha, Gorakshakar Ajit

**Affiliations:** a Department of Transfusion Medicine, ICMR-National Institute of Immunohaematology, KEM Hospital Campus, Maharashtra, India; b Department of Hematogenetics, ICMR-National Institute of Immunohaematology, KEM Hospital Campus, Maharashtra, India; c ICMR-National Institute of Immunohaematology, KEM Hospital Campus, Maharashtra, India

**Keywords:** ACKR1, PCR-RFLP, In-frame insertion, Agri, In silico

## Abstract

**Introduction:**

Atypical chemokine receptor 1 (*ACKR1*) which carries the Duffy antigens, is not just a blood group antigen but serves many more functions. It is a receptor for various pro-inflammatory and inflammatory chemokines and for *Plasmodium vivax*. Genetic variations in *ACKR1* are the basis for Duffy blood group antigens.

**Methods:**

Routine serological Fy^a^/Fy^b^ typing and *ACKR1* genotyping by Polymerase Chain Reaction*-*Restriction Fragment Length Polymorphism was employed in a population study that included 331 samples from the Agri community.

**Results:**

Weak Fy^b^ expression was detected by serological findings in five unrelated samples, which prompted further investigation by molecular means. By Polymerase Chain Reaction an aberrant pattern was demonstrated on polyacrylamide gel electrophoresis, which led to the identification of an alteration by sequence analysis. This study describes a 3-bp insertion, present in the FY*B allele (c.144_146dupTGC), resulting in the insertion of the amino acid alanine (p.A49dup) within the full-length protein.

**Conclusion:**

The 3-bp in-frame insertion (c.144_146dupTGC, p.A49dup) (rs765671589) in the *ACKR1* gene was identified in five individuals from the Agri community. Despite apparently carrying an FY*B allele, a very weak Fy^b^ antigen expression was found in association with this genotype. This insertion may also have implications for some physiological roles of *ACKR1* and be of interest in malaria research and population genetics.

## Introduction

Atypical chemokine receptor 1 (*ACKR1*) [1] is a multi-pass trans-membrane protein found on the erythrocyte surface that is involved in the invasion of red blood cells (RBCs) by *Plasmodium vivax* (*P. vivax*) merozoites [2]. It is considered one of the most paradigmatic examples of positive selection in the human genome due to its strong geographic differentiation and relationship with resistance to vivax malaria [2]. It is a G protein-coupled receptor (a 35–50 kDa glycoprotein) found on the surface of RBCs and endothelial tissue [3]. ACKR1 is also expressed in erythroid progenitor cells of bone marrow, where *P. vivax* invasion may occur [4,5]. *ACKR1* expresses the antigens of the Duffy blood group system where the major alleles are FY*A (FY*01) and FY*B (FY*02), which are co-dominantly expressed.

The Duffy blood group system is clinically significant in transfusion medicine because antibodies against its antigens may cause haemolytic transfusion reactions (HTR) and haemolytic disease of the newborn (HDN) [3,6]. *FY* (FY*A and FY*B) alleles differ by a single base change at nucleotide c.125 due to a *G* > *A* substitution (rs12075) [3]. The resultant antigens differ by a single amino acid at the 42nd position that encodes glycine in (Fy^a^) and aspartic acid in (Fy^b^) (D42G). This amino acid change does not affect chemokine binding affinity [7]. Anti-Fy^a^ and anti-Fy^b^ antisera allow detection of the four main phenotypes: Fy(*a* + *b*+), Fy(*a* + *b*-), Fy(a-*b*+), and Fy(a-b-) [6,8]. Although the Fy(a-b-) phenotype is the dominant phenotype in black human populations, particularly those of West African descent, it is rare in non-black people [8,9]. Most West Africans and two-thirds of Afro-Americans do not express ACKR1 on the surface of RBCs resulting in the Fy(a-b-) phenotype [3,10, 11, 12]. The absence of ACKR1 from the RBC surface is due to a homozygous substitution (c.–67T>*C*) (rs2814778) in the 5′ untranslated region of the *FY* gene, also known as the *GATA-1 box* (FY*02 N.01).

The Fy^x^ phenotype is caused due to the polymorphisms at nucleotide c.265 *C* > *T* (rs34599082) and at nucleotide c.298 *G* > *A* (rs13962) [12, 13, 14]. These nucleotide changes reduce Fy^b^ antigen expression to the extent that only a few anti-Fy^b^ reagents detect the antigen by haemagglutination. Weak Fy(*b* + *^w^*) [Fy^x^] expression has also been linked to the loss of a nucleobase C in the sequence spanning the regulatory element *Sp1* site [15]. Other polymorphisms in the *ACKR1* gene that silence *FY* [or a Fy(a-b-)] phenotype include a 14-nucleotide deletion in Fy^a^, which causes a frameshift and a premature stop codon [16], and three separate nucleotide changes, which are responsible for converting the Trp codon found at different positions to a stop codon in either FY*A or FY*B alleles [16,17].

The distribution of the ACKR1 allele FY*02 *N.01* has been used to assess the admixture and ethnicity of various populations, e.g., Saudi Arabs, Israeli Jews, Njazidia, and descendants of Africa [18, 19, 20, 21, 22]. Characterizing the pattern of genetic variations among numerous ethnic populations is vital for constructing human evolutionary records, investigating population records, and evaluating the right design and acumen of genetic disease affiliation studies [23,24].

Genetic variations among different communities in India are opaque. There exists vast human diversity in India, with more than four thousand anthropologically well-defined populations, each differing in language, culture, customs, and genetic makeup [25]. Amongst them, one of the populations is the Agri community. This community is seen mainly in Thane, Raigad and Palghar districts of Konkan division, Maharashtra, India. They are mainly engaged in farming, fishing, and salt making. Their population in India is estimated to be about 524,000, and they are reported to be found only in India [25]. They consider themselves just below Brahmins and Kshatriyas in the Indian caste hierarchical system. The Agris is subdivided into several divisions (*snull kul*), which regulate marriages. Exogamous marriages are allowed, but within the same *snull kul*, consanguineous marriages are not allowed [26]. Inter-community weddings are rare in Agris.

While studying the occurrence of *ACKR1* gene polymorphisms in the Agri community, a 3-bp insertion (c.144_146dupTGC) insertion (rs765671589) was identified. This in-frame insertion is predicted to insert Alanine at position 49 of the major isoform of the ACKR1 protein. ACKR1 expression on RBCs may be decreased or absent due to this nucleotide change in the *FY* gene. This example is remarkable with a 3-bp in-frame insertion (c.144_146dupTGC) to be documented in an Indian tribal population with serological data to support the effect of alteration on Duffy antigen stability. This study highlights the importance of establishing the incidence and nature of molecular events that could impact ACKR1 antigen expression in Agri.

## Materials and methods

### Study population

A total of 331 samples from the Agri community were collected during various camps organized by Indian Council for Medical Research –National Institute of ImmunoHematology (ICMR-NIIH) in Navi-Mumbai, Maharashtra to investigate the incidence of different red cell antigens. Unrelated males and females aged over 18 years participated in this study. Information on the patient’s ethnic background, presence of other diseases such as diabetes, infectious disease (including malaria), and smoking were obtained by interviewing the individuals.

### Ethical considerations

Detailed information was provided and explained about the research to be carried out on their blood sample to all the individuals who agreed to participate in the study. All studies were performed according to the recommendations put forth by the Institutional Ethics Committee for Research on Human Subjects, National Institute of Immunohaematology (ICMR), Mumbai.

### Blood collection

Peripheral blood was collected (3 mL in EDTA and 4 mL in plain vacutainers) and stored at 4 °C till samples reached the Institute. The collected blood was used for serological and molecular typing of various blood group antigens including Duffy.

### Serological testing

Duffy phenotyping was determined by haemagglutination assay using a monoclonal antibodies Anti-Fy^a^and Anti-Fy^b^ (Gamma-clone); Cat.: 3013–2; Immucor, Inc. Norcross, GA, USA) by the direct tube method and gel cards (Diamed SA, Morat, Switzerland) according to manufacturer’s recommendations. Suitable controls were included during the serological phenotyping of RBCs.

### DNA extraction

The phenol-chloroform method was employed to isolate genomic DNA from the peripheral blood [27]. DNA quantity and quality were assessed by Nanodrop-1000 (Thermo Fisher Scientific, Massachusetts, US), and the samples were diluted to attain a final concentration of 30 ng/µL.

### Polymerase chain reaction-restriction fragment length polymorphism genotyping

*ACKR1* polymorphisms which included promoter *GATA-1 box* c.–67T>*C* (FY* Null (FY*02 *N.01*), nucleotide changes c.125 *G* > *A* (FY*01/FY*02), c.265 *C* > *T*, and c.298 *G* > *A* (FY*01 W.01 or FY*02 *W.01*) were identified by polymerase chain reaction followed by restriction fragment length polymorphism (PCR–RFLP) as described by Castilho et al. [28] with minor modifications which included the choice of primer sequence (Table 1) and restriction enzymes (Table 2).

**Table 1:** Primer sequences flanking *ACKR1* and *GATA-1* and three Single Nucleotide Polymorphisms.

Name	Sequence (5′ → 3′)	PCR Product Size (bp)	T_m_ ( °C)	T_a_ ( °C)
ACKR1_FP	CATGGCACCGTTTGGTTCAG	189	61.3	58.1
ACKR1_RP	CAAGGCCAGTGACCCCCATA			
FY_AB_FP	TCCCCCTCAACTGAGAACTC	392	58.2	55.3
FY_AB_RP	AAGGCTGAGCCATACCAGAC

**Table 2:** Polymerase Chain Reaction*-*Restriction Fragment Length Polymorphism (PCR-RFLP) genotyping of ACKR1 antigens using different restriction enzymes.

rs Number	Amino Acid Change	Restriction Enzyme	Genotype	Digestion	PCR Restriction Fragment Lengths (-bp)
rs2814778 (c.−67T>*C*)	-	*Sty I*	T/T	Undigested	189
			T/C	Digested	189, 108,81
			C/C	Digested	108, 81
rs12075 (c.125G>*A*)	G42D	*Ban I*	A/A	Undigested	392
			A/G	Digested	86, 94, 212, 306
			G/G	Digested	86, 92, 212
rs13962 (c.298G>*A*)	A100T	*Mwo I*	A/A	Undigested	392
			A/G	Digested	51, 67, 274, 341
			G/G	Digested	51, 67, 274
rs34599082 (c.265C>*T*)	R89C	*Aci I*	G/G	Undigested	392
			C/G	Digested	156, 236, 392
			C/C	Digested	156, 236

Polymerase chain reaction (PCR) was performed using 150 ng of DNA, 5 pmol of each primer, 2.5 nmol of each dNTP, 1.0 U Taq polymerase, and buffer (Genei Labs, Bangalore, India), a total volume of 25 µL. Two sets of primers were used for genotyping *ACKR1* variants. The first set of primer pair (ACKR1_FP and ACKR1_RP, Table 1) flanked c. −67 *T* > *C GATA-1* of the erythrocyte-specific transcription factor eTFII. The second primer pair (FY_AB_FP and FY_AB_RP) spanned the *ACKR1* gene region containing the remaining three polymorphic sites.

PCR was performed in a thermal cycler (S-96 Gradient Thermal Cycler, Quanta Biotech, USA) with the cycling conditions as 95 °C for 5 min, followed by 35 cycles [95 °C for 45 s; annealing at X °C (Table 1) for 45 s; elongation at 72 °C for 45 s]; final extension of 5-min incubation at 72 °C; and 4 °C 5-min. The PCR products were loaded on 1 % agarose in Tris-acetate-EDTA (TAE) buffer and were electrophoresed for 35 mins at 80 V to check for amplification efficiency before treatment with restriction enzymes as per the manufacturer’s instruction. The digested products were run on 12 % polyacrylamide gel. The restriction digestion patterns of the PCR products (in base pairs) with specific enzymes are enlisted in Table 2.

### DNA sequencing

The polymorphic sequence within exon 2 was amplified by PCR using FY_AB_F and FY_AB_R primers (Table 1). The amplified PCR product was cleaned using ExoSAP-IT (USB Corporation, Cleveland, Ohio) and then sequenced using 3700XL automated DNA sequencer (Applied Biosystems, USA). Sequencing was performed using the fluorescent Big-Dye Terminator v.1.1 cycle sequencing kit (Applied Biosystems, Foster City, CA, US) as per the manufacturer’s protocol. The National Center for Biotechnology Information reference sequence used for *ACKR1* was NM_002036.2. Mutation surveyor analysis tool was used to analyse the raw data obtained from DNA sequencer.

### NEBcutter V2.0

The online tool NEBcutter V2.0 was used to assess if the insertion of 3-bp in the coding sequence (sequence flanked by FY_AB_FP and FY_AB_RP primers), generates aberrant band size on gel when digested by restriction enzyme *Ban I*.

### In silico analysis

PROVEAN (Protein Variation Effect Analyzer) is an online software tool that predicts the impact of nucleotide substitution or indel on the protein's functionality. The nonsynonymous or indel variants that are expected to have functional significance can be found using PROVEAN. PROVEAN uses the score thresholds for prediction (deleterious or neutral). The default threshold value is set to −2.5. Variants with scores equal to or below −2.5 were considered “Deleterious,” while the variants with a scores above −2.5 were considered “Neutral.” For our analysis, the variant score was generated by comparing 215 sequences and constructing 30 clusters. (link: https://www.jcvi.org/research/provean) (supplementary data)

## Results

### Serology

**Fig. 1:**
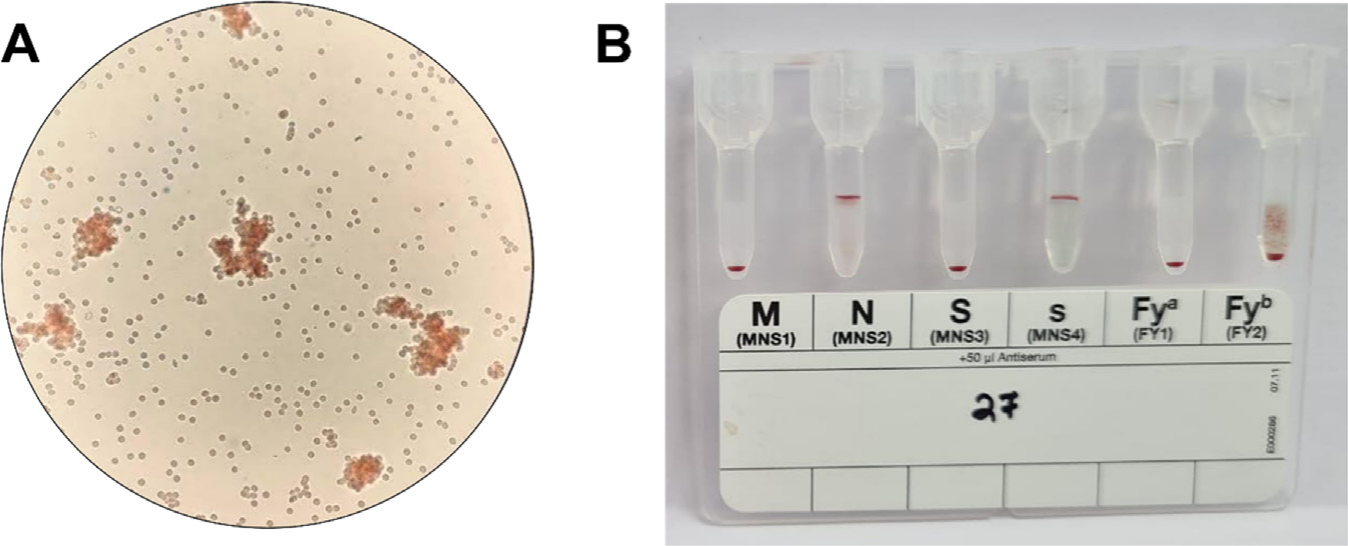
Serological typing of ACKR1 antigen. A. Standard serological typing of ACKR1 antigen using tube method. Weak to very weak agglutination reaction was observed under (X10) microscopic field. B. Validation of tube method of ACKR1 investigation using Diamed gel cards showing no agglutination reaction (Fy^a^) and weak agglutination reaction (Fy^b^).

Three hundred and thirty-one samples were tested using FY monoclonal antibodies by standard tube technique. Of these, 326 samples showed either Fy(*a* + *b*-), Fy(a-*b*+), or Fy(*a* + *b*+) phenotypic distribution. FY null [Fy(a-b-)] was not found in the tested population. Five samples showed weak to very weak (+) agglutination reactions with anti-Fy^b^ for Fy^b^ (Figure 1A); however, when tested on gel cards, it was found to be a mixed field (mf) reaction type (Figure 1B); which prompted us to investigate these samples further using molecular analysis.

### Polymerase chain reaction-restriction fragment length polymorphism

**Fig. 2:**
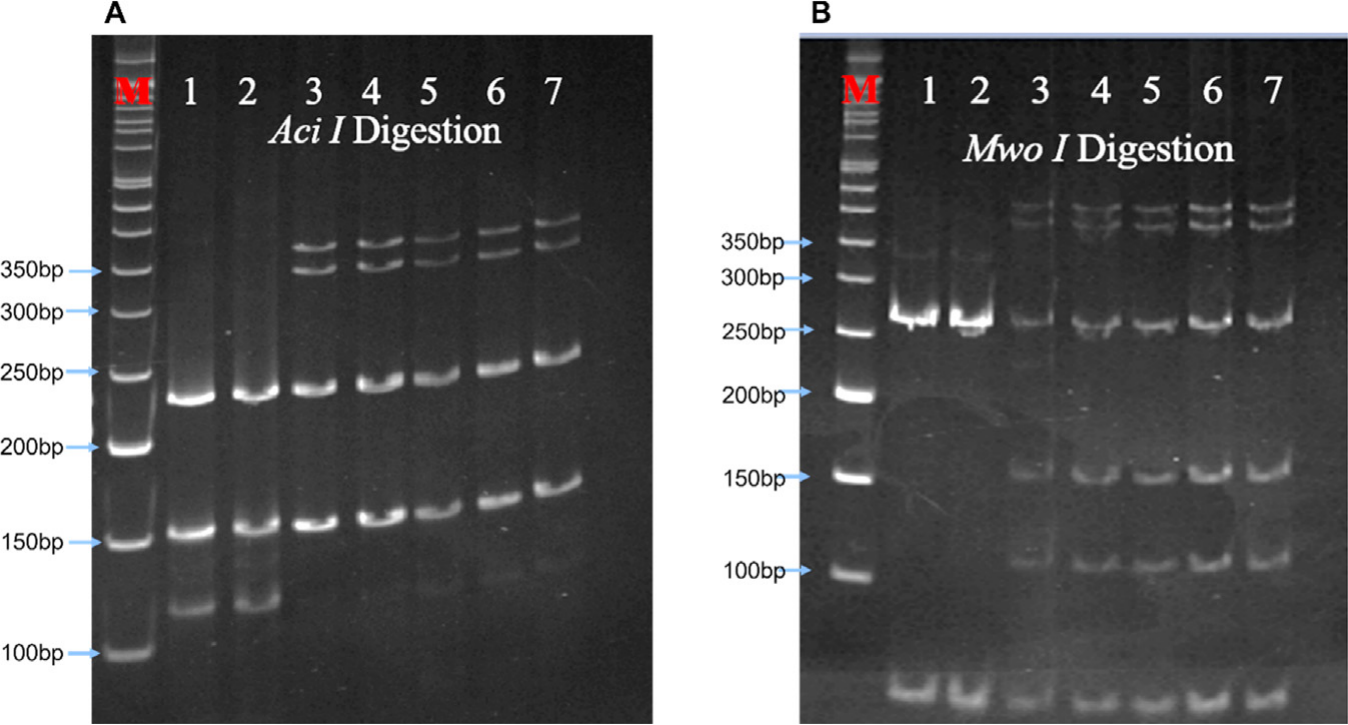
Polyacrylamide gel electrophoresis. Aberrant band pattern separation of restriction enzymes A. *Aci I* and B. *Mwo I* digested polymerase chain reaction (PCR) product on 12 % polyacrylamide gel. The gel picture showed the restriction digestion patterns of the PCR products from samples under investigation (samples 3, 4, 5, 6, and 7) and samples with known genotypes, which were used as the digestion and migration controls (samples 1 and 2). *Aci I* digestion produced two fragments viz. 156-bp and 236-bp, while *Mwo I* produced three fragments of sizes 51-bp, 67-bp and 274-bp. M: 50 bp DNA ladder (Cat. No.: DM012-R500, GeneDirex). The gels were stained with ethidium bromide (0.5μg/mL final concentration in 0.5x TBE buffer) for 15 mins with gentle shaking, followed by destaining for 20 mins with deionized water.

All five analysed samples produced 189-bp products using ACKR1_FP and ACKR1_RP primers, which on digestion with *Sty I* produced a product of size viz. 189-bp, indicating the presence of wild-type T at c. −67 *T* > *C* (*GATA-1*) position. The five amplified samples using the primers FY_AB_F and FY_AB_R were genotyped as Fy(a-*b*+) using *Ban I* restriction enzyme. The aberrant band migration patterns observed post-digestion with *Aci I* and *Mwo I* enzymes were characterized by slight shifts in the electrophoretic mobility of the restriction fragments compared to control samples. Specifically, the 156-bp and 236-bp fragments from *Aci I* digestion and the 274-bp fragment from *Mwo I* digestion showed altered migration (Figure 2) distances due to the 3-bp in-frame insertion that modified the overall conformation of the DNA fragments, despite not creating or destroying restriction sites. These subtle but consistent mobility differences prompted further sequence analysis to identify the exact nature of the genetic variation.

### DNA sequencing

**Fig. 3:**
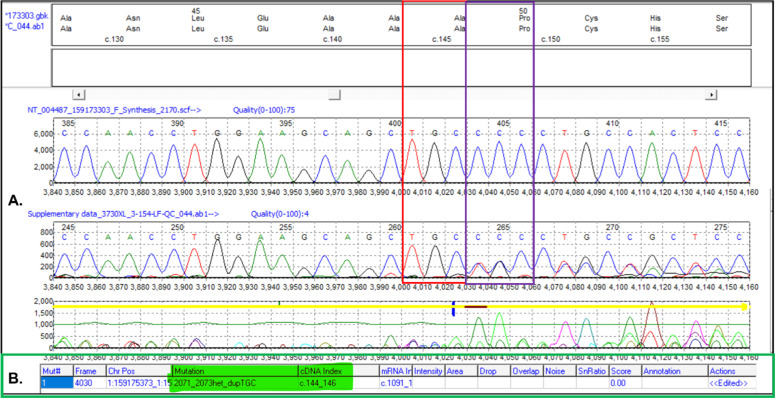
Electrophoretogram of *ACKR1* sequence. A. Comparison of electrophoretogram of polymerase chain reaction (PCR) product from a control sample (wild-type sequence) and sequence with 3-bp insertion (altered sequence). B. The result out-put generated by Mutation Surveyor shows insertion of three base pairs (TGC) in *ACKR1* sequence.

The five samples with aberrant band patterns were taken up for Sanger sequencing to delineate the sequence changes. Mutation surveyor analysis tool was used for electrophoretogram analysis which showed the insertion of three bases TGC [c.144_146dupTGC; c.144_146dup] (Figure 3) in *ACKR1* CDS when compared to the reference sequence (NM_002036.2). This 3-bp in-frame insertion resulted in the insertion of an amino acid Alanine (A49dup) (Figure 3). The 3-bp insertion resulted in an increment of an amino acid to 337 as against wild type with 336 amino acids (Supplementary Figure 1). This variant was deposited in the dbSNP database under reference Single Nucleotide Polymorphism (SNP) cluster ID: **rs765671589**. However, neither publication report nor clinical significance is available pertaining to the **rs765671589** on the dbSNP website. The tested sample also did not show presence of any other known variant other than c.144_146dupTGC. The aberrant pattern observed on acrylamide gel might be due to the in-frame insertion of three bases. It is important to emphasize that the 3-bp insertion in the tested samples was in heterozygous state.

### In silico analysis

*In silico* analyses using the online platform PROVEAN predicted the three-base insertion to be deleterious with a score value of −5.021 (cut-off = −2.5) (Supplementary Figure 2 and supplementary data). NEBcutter V2.0 analysis showed that the insertion of 3-bp did not alter the recognition and restriction sites of the enzyme.

## Discussion

This paper describes a 3-bp in-frame insertion (c.144_146dupTGC, p.A49dup) identified in five individuals (probably unrelated) of Agri descent who were identified to be FY*B homozygous. The RBCs from these individuals showed weak to very weak (+) agglutination reactions with anti-Fy^b^, indicating that the reduction of Fy^b^ antigen on their surface produced a visible, very weak haemagglutination reaction under the microscope. We hypothesize that the altered ACKR1 protein (p.A49dup) might: 1) not be well integrated on the RBC membrane, 2) be inefficiently transported to the membrane, or 3) be substantially degraded before being transported on RBCs membrane. The former events may affect the detection of the Fy^b^ antigen on RBCs by commercially available antisera.

The insertion of Ala at amino acid 49 position in the extra-cellular segment of Fy^b^ is predicted to be deleterious by the *in silico* platform PROVEAN. Alanine is an ambivalent and non-polar amino acid with an optically active chiral C atom. It has a *β*-carbon (methyl group), which hinders conformation changes that the backbone can adopt. Being a hydrophobic amino acid, alanine contributes to closeness in protein folding by repelling water. The presence of extra alanine residues might mitigate the structural integrity.

The expression pattern observed in this study bears similarities to findings reported by Parasol et al. [12], who described a novel change in the FY*B allele leading to an altered erythrocyte phenotype, though in their case it was due to a *C* > *T* substitution at nucleotide 265. Similarly, Tournamille et al. [13] reported that an Arg89Cys substitution results in very low membrane expression of the Duffy antigen/receptor for chemokines in Fy^x^ individuals. Our findings represent a different molecular mechanism (insertion rather than substitution) leading to reduced Fy^b^ expression, adding to the spectrum of known Duffy-related variations with phenotypic consequences.

It is important to note that the insertion described here is in a heterozygous state, and the effect of this alteration needs to be addressed in individuals with homozygous insertion, providing a piece of concrete evidence on protein stability on RBCs. We made attempts to approach the individuals; however, due to unavoidable situations, we were not able to collect specimens from family relatives, keeping this avenue to be explored in the future.

Genotyping of blood group antigens, including Duffy, has been used for decades to assess population admixture and ethnic backgrounds. *ACKR1* polymorphisms are characterized in African populations, and the identity of the *Fy^bES^* variation in non-African populations has been considered a probable situation for the admixture of African–American [12,28,29]. Consequently, our data suggest that c.144_146dupTGC, p.A49dup in *ACKR1* may be peculiar to the Agri community. The data does not confirm the racial identity specific to the community. Even though the observation provides insight that this alteration may be specific to this community, we need to establish/prove this hypothesis. The Agri community comprises people who prefer marriage within the community, and this endogamy might be the reason for the observed higher occurrence of this variation in this specific community.

The identification of a novel 3-bp insertion (c.144_146dupTGC; rs765671589) in the *ACKR1* gene within the Agri community represents a significant molecular discovery, particularly given its absence in published literature despite being catalogued in the dbSNP. Our documentation of this insertion provides the first comprehensive characterization of rs765671589 in a specific population, contributing valuable data to the growing landscape of *ACKR1* polymorphisms. This finding has multifaceted implications: it enhances our understanding of genetic diversity in minority populations, potentially influences individual and population-level disease susceptibility profiles, and provides crucial insights into regional genetic adaptations. Furthermore, this discovery opens new avenues for investigating the functional consequences of *ACKR1* modifications and their potential impact on receptor expression, chemokine binding affinity, and disease associations in the Agri community. The presence of rs765671589 (c.144_146dupTGC, p.A49dup) might also have implications for managing transfusion therapy in this community.

The FY*B product is often difficult to detect using commercially available anti-Fy^b^ reagents used in routine RBC typing. Often Fy^x^ identification by anti-Fy^b^ goes undetected, and such samples are reported as negative. This is true even when the tests are repeated under the same conditions [30]. It is recommended to type RBCs using PCR/PCR-RFLP-based methods to distinguish Fy^x^ samples from that of Fy null [Fy(a-b-)] instead of employing several anti-Fy^b^ sera or a labour-intensive and cumbersome adsorption and elution technique. William et al. showed that ACKR1 antigens weaken on storage at 4 °C [31]. It is, thus, suggested to phenotype the samples for ACKR1 antigens immediately without delay.

Gene alternations conferring malaria resistance are often under balancing selection, maintaining deleterious alleles at high frequencies [31]. Erythrocyte polymorphisms, strongly shaped by malaria [32,33], affect structural proteins or metabolic enzymes, limiting parasite growth [34]. These polymorphisms are prevalent in malaria-endemic regions [35]. King et al. demonstrated the higher affinity of Fy^b^ for *P. vivax -*ACKR1 binding protein compared to Fy^a^ [36]. The p.A49dup insertion, predicted to be deleterious *in silico*, may protect against malaria by affecting Fy^b^ stability. Further research is needed to quantify its protective effect compared to Fy^a^ through binding and inhibitory assays, particularly for *P. vivax* infections.

Our study provides valuable insights into the c.144_146dupTGC (rs765671589) variant in the *ACKR1* gene, though there are several areas where future research could further enhance our understanding. While we successfully characterized the variant in heterozygous individuals, the identification and analysis of homozygous cases would provide additional insights into its full impact on ACKR1 protein expression and function. Although we made considerable efforts to collect family samples from the identified cases, unforeseen circumstances prevented this extension of our study, presenting an opportunity for future family or community-based investigations. The study opens several promising avenues for further research, including 1. Expanded molecular characterization using quantitative protein expression analysis to complement our current serological findings. 2. Additional functional studies to explore the variant's potential effects on chemokine binding and *P. vivax* interactions. 3. Broader geographical sampling within the Agri community to better understand the variant's distribution pattern. 4. Longitudinal follow-up studies to observe Fyᵇ expression patterns over time, if possible. 5. Extended family studies to better understand the inheritance patterns of this variant within the community. These research opportunities could provide valuable additional context to our findings and further illuminate the role of this variant in *ACKR1* function. Our current results lay a strong foundation for such future investigations, which could build upon the molecular and serological characterization we have established.

## Conclusion

In summary, we report five individuals with the FY*02 allele carrying c.144_146dupTGC, p.A49dup in exon 2 of the *ACKR1* gene, with significantly reduced Fy^b^ expression on the RBCs surface. This in-frame insertion, resulting in p.A49dup, has not been previously described. The in-frame change identified may not be directly related to the exposure of a human genome to *P. vivax*. However, it may have arisen due to *de novo* insertional alteration specific to the Agri community. However, this hypothesis needs further supporting data. Identifying rs765671589 genotypes in *FY* will provide clues for better genotype-phenotype correlation and may aid in explaining the molecular pathogenesis in diseases and in population genetics. These discoveries feature the significance of different investigations for better comprehension of the genetic basis of blood group antigens.

## Author contributions

S.R. conducted the experiment. S.P. provided technical help. G.A. and G.K. supervised the experiments. S.R. conducted data analysis. G.A. conceptualized the project, was responsible for the overall supervision, and procured funding. S.R. wrote the manuscript. G.A. and G.K. approved the final manuscript.

## Data availability statement

The data that support the findings of this study are available on request from the corresponding author.

## Generative AI and figures, images and artwork

The authors did not employ generative AI or AI-assisted technologies to write the manuscript and take full responsibility for the content of the publication.

## Funding

The study was funded by the Indian Council of Medical Research. The funder had no role in the study design, data collection and analysis, decision to prepare or to publish the manuscript.

## Conflicts of interest

The authors report no conflicts of interest.

## Appendix Supplementary materials

Image, application 1
