# Supplementary material for: Identification of an in-frame insertion in ACKR1 in five individuals from Agri community, India
Source: Hematol Transfus Cell Ther. 2025 Oct 30;48(1):106075. doi: 10.1016/j.htct.2025.106075 (PMC12613020; doi:10.1016/j.htct.2025.106075)
Supplement: Supplementary file 1 [file mmc1.docx]

**Supplementary Figure 1:**

**
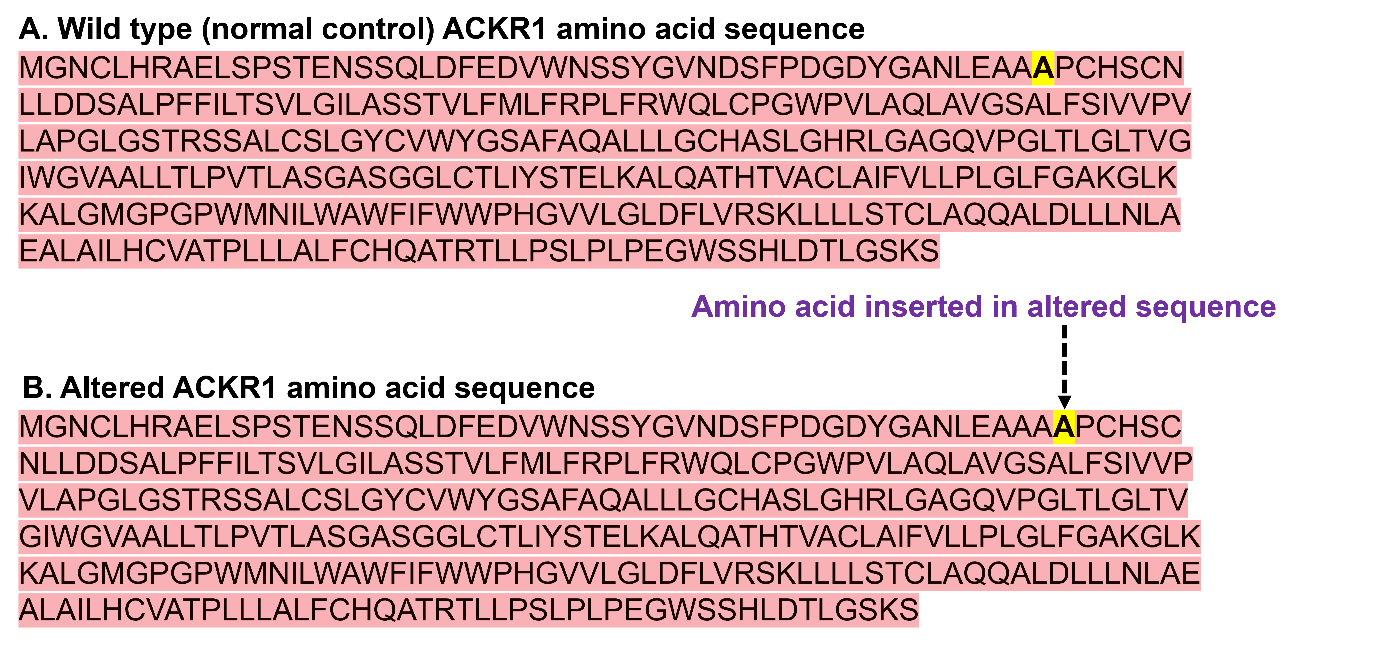
**

**Supplementary Figure 2:**

**
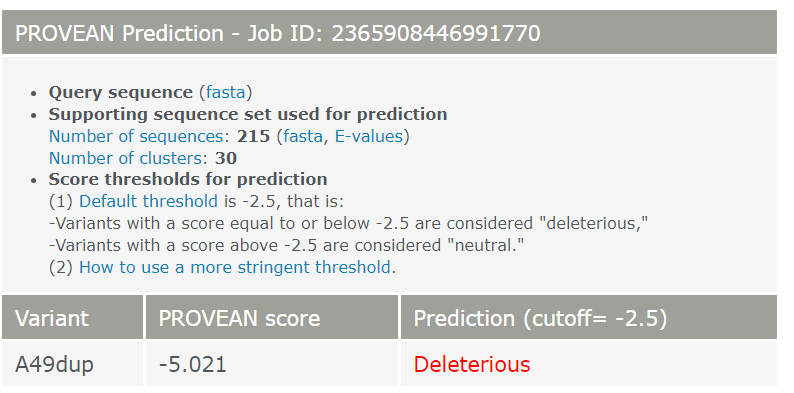
**

**Supplementary Figure 1: Alignment of ACKR1 antigen (amino acid) of wild-type and altered sequences.** The upper shows amino acid sequences of wild-type (control) ACKR1 antigen. The lower panel shows ACKR1 antigen with an altered sequence. The yellow bold amino acid A highlighted in the sequence is inserted in the sequence. The online Expasy translate tool was used to deduce the amino acid sequence from wild-type and altered *ACKR1* nucleotide sequences (ACKR1(NM_002036.4):c.144_146dup)

**Supplementary Figure 2: *In silico* analysis of altered amino acid sequence by online plate forms. A.** Protein prediction by PROVEAN. The single amino acid deleted variant was predicted to be deleterious. **B.** Virtual gel electrophoresis generated by NEBcutter V2.0. **a**: Gel image generated upon digestion of wild type sequence with *Ban I*; **b**: Gel image generated upon digestion of variant sequence with *Ban I*. The horizontal red line helps to illustrate the difference in band size on the gel.
